# Advanced Characterization of Solid-State Battery Materials Using Neutron Scattering Techniques †

**DOI:** 10.3390/ma17246209

**Published:** 2024-12-19

**Authors:** Eric Novak, Luke Daemen, Niina Jalarvo

**Affiliations:** 1 Department of Physics and Astronomy, Swarthmore College, 500 College Ave, Swarthmore, PA 19081, USA; 2 Neutron Sciences Directorate, Oak Ridge National Laboratory (ORNL), Oak Ridge, TN 37831, USA; daemenll@ornl.gov

**Keywords:** solid-state battery, materials research, neutron scattering, neutron diffraction, inelastic neutron scattering, quasielastic neutron scattering, neutron reflectometry, small-angle neutron scattering, neutron imaging, interfaces

## Abstract

Advanced batteries require advanced characterization techniques, and neutron scattering is one of the most powerful experimental methods available for studying next-generation battery materials. Neutron scattering offers a non-destructive method to probe the complex structural and chemical processes occurring in batteries during operation in truly in situ/in operando measurements with a high sensitivity to battery-relevant elements such as lithium. Neutrons have energies comparable to the energies of excitations in materials and wavelengths comparable to atomic distances in the solid state, thus giving access to study structural and dynamical properties of materials on an atomic scale. In this review, a broad overview of selected neutron scattering techniques is presented to illustrate how neutron scattering can be used to gain invaluable information of solid-state battery materials, with a focus on in situ/in operando methods. These techniques span multiple decades of length and time scales to uncover the complex processes taking place fundamentally on the atomic scale and to determine how these processes impact the macroscale properties and performance of functional battery systems. This review serves the solid-state battery research community by examining how the unique capabilities of neutron scattering can be applied to answer critical and unresolved questions of materials research in this field. A thorough and broad perspective is provided with numerous practical examples showing these techniques in action for battery research.

## 1. Introduction

The ongoing transition to cleaner renewable energy resources presents an ever-growing demand for high-density, next-generation energy storage materials. The electrification of transportation, portable electronic devices, and grid-scale energy storage will all require advanced batteries. There is a clear need to improve existing energy storage materials to meet this demand, as well as discover new, better performing technologies. While liquid electrolyte-based batteries continue to dominate the rechargeable battery market, designs based on solid electrolytes are promising candidates for use in advanced batteries that offer increased safety, faster charging, and enable the use of high energy density anodes and high voltage cathodes [1,2,3,4]. Despite the intriguing potential, technical barriers are still limiting the practical implementation of solid-state batteries [5,6,7]. Advanced materials characterization techniques are therefore needed to provide new insights into the complex chemical processes that are occurring and evolving dynamically during active battery operation. The experimental techniques need to span multiple orders of magnitude of length and time scales to gain a complete understanding of how the atomic-scale phenomena influence the bulk-scale battery properties and performance [8,9,10]. The ideal probe would be non-destructive and allow for truly in situ and in operando measurements of a battery during electrochemical cycling. One of the best methods available that can accomplish these tasks is neutron scattering [8,9,11,12,13,14,15]. Unfortunately, neutron scattering remains a specialized experimental tool that is largely underutilized in the scientific community. This review is intended to demonstrate the important role that neutron scattering can play in advancing the current knowledge of solid-state energy storage materials and to show the vast amounts of information that can be gained through neutron scattering experiments on these systems, much of which cannot be achieved with any other experimental technique.

Li-based liquid electrolyte batteries have long dominated the battery industry due to their high energy density and long cycling lifetime. However, limited Li reserves and high demand are driving up the cost, a feature that is unlikely to improve going forward [16]. While Li-based batteries will continue to assume their key role in the future, it is necessary to develop alternative battery sources not based on Li (e.g., Na, K, Al, Zn, and Mg) [14,17]. In addition, liquid-based electrolytes are prone to leakage, flammability, and dendrite formation (short circuit cell failure). Solid-state materials can prevent dendrite formation and offer high mechanical and thermal stability, hence providing a potentially safer electrolyte compared to their liquid analogues [2]. The solid-state electrolyte is a crucial component of efficient devices since it provides not only an ionic diffusion pathway but also serves as a separator between the cathode and anode. The interphase that forms between the solid electrolyte and the electrodes is a critical region for ensuring high performance and long lifetimes. While great advancements have been made, solid-state batteries continue to face some challenges, such as obtaining fast ionic transport at reasonable operating conditions, high interfacial impedance, maintaining proper contact between components during cycling, and producing solid electrolytes that are chemically and thermodynamically stable at voltages relevant for high-performance devices [1,2,3,7,18,19,20]. Solutions to these issues can only be found if there is a fundamental understanding of the atomic-scale mechanisms that take place during the operation of the battery. With this information, the synthesis can be better directed and controlled to produce tailored properties for advanced performance. A key tool for gaining this understanding is neutron scattering techniques.

Comprehensive overviews of neutron scattering theory have been presented previously [21,22,23,24]. Neutron scattering and light scattering techniques are similar in many ways, but neutrons possess several key advantages over their photon analogues. This is because neutrons are scattered by atomic nuclei while photons are scattered by electrons. For example, X-ray diffraction (XRD) measurements are unable to readily resolve the signal from low atomic number elements in the presence of heavier nuclei since the scattering potential scales with the number of electrons. This can be problematic for battery research since many of the elements of interest are light elements, i.e., H, Li, C, O, etc., which means that some battery components, such as the electrolyte, SEI, and Li-rich regions, will be almost invisible to X-ray techniques. On the other hand, since neutrons interact with the atomic nuclei, the scattering potential does not scale with atomic number, and neutrons are actually highly sensitive to light elements and can even distinguish between different isotopes, which cannot be accomplished with X-rays. Another benefit is that neutrons are not governed by the selection rules of light scattering, which allows all vibrational modes to be active. Perhaps the biggest advantage for battery research is the lack of charge on the neutron, which allows for deep sample penetration to probe the bulk properties. This is advantageous because it can probe all the cell components simultaneously to allow truly in situ/in operando measurements of batteries. This overcomes the need for opening the battery and hence disturbing the cell chemistry and structure, which is a common technique employed in ex situ studies. Neutron scattering also does not distribute heat in the sample, a feature that would otherwise cause uneven electrochemical reactions. However, this is not always the case for X-rays, and great care should be taken for these types of measurements. For example, the high energies and flux offered at synchrotrons can damage battery components, especially organic materials such as polymers, electrolytes, and the polymeric nature of some SEIs. Blondeau et al. reported that operando synchrotron X-ray measurements were invalidated due to modification of the electrodes and electrochemical processes caused by the X-ray irradiation [25].

Neutron scattering simultaneously probes both the structure and dynamics of materials, yielding not only the positions of atoms in a material but also how they are moving (e.g., diffusion, phonons, molecular vibrations). For a neutron interacting with an array of atoms, the total scattering potential is a sum of two forms of scattering: coherent and incoherent. Coherent scattering occurs when the interaction causes strong interference of the scattered wave. This scattering condition is satisfied when the momentum transfer, *Q*, corresponds to a reciprocal lattice vector. Coherent scattering therefore provides information about the spatial arrangement of atoms in the material. This structural information is collected in a neutron diffraction experiment by measuring the elastic (no energy transfer) scattering signal. On the other hand, the incoherent cross section lacks this phase relationship and instead contains information about single particle dynamics. Rather, it measures a correlation between the position of a particle at the origin, *R*(0), compared to the position of the particle at a later time, *R*(*t*). Incoherent scattering provides information about both the temporal and spatial motion of the atoms in the sample to probe motions such as diffusion. These motions are measured using quasielastic and inelastic neutron scattering since the motion involves a transfer of energy.

Existing review articles covering neutron scattering techniques for battery research have been mostly focused on specific materials, e.g., batteries based on Li [9,10,11,13,15] and Na [14,26], or the review focuses on a specific neutron technique [27,28,29,30]. Some studies also compare the use of neutron scattering techniques in combination with other advanced probes such as muon sources, electron scattering, optical techniques, and synchrotron X-ray scattering for battery research [12,31]. Our work presents a broad, suite-wide overview of neutron scattering techniques with a focus on solid-state batteries and in situ/operando methods. Each technique is introduced with a brief theoretical background and followed with scientific examples and numerous references to help the reader to obtain a comprehensive picture of the contribution that neutron scattering can make to the solid-state battery research and development. Our review also contains a neutron spectroscopy section, which is a technique that holds great potential for battery materials research, but apart from QENS electrolyte studies, it is not a main area of focus for battery research and hence is not a focal point of discussions in the previous reviews. Specifically, we will highlight experimental results from inelastic neutron scattering (INS), quasielastic neutron scattering (QENS), small-angle neutron scattering (SANS), neutron reflectometry (NR), neutron imaging (NI), neutron powder diffraction (NPD), total neutron scattering, and pair distribution function (PDF) analysis.

This review covers three broad sections of neutron scattering for battery research: (1) structural investigations, (2) studies of interfaces and bulk-scale components, and (3) dynamics. Each technique, as well as individual instruments, is purposely designed to have distinct functionalities and excel at measuring specific regimes. Neutron spectroscopy probes the dynamics to study ionic diffusion and lattice dynamics. Diffraction yields crystallographic information, while total neutron scattering (PDF analysis) can be used to probe amorphous and disordered structures, which may be used in next-generation devices [32,33]. SANS, NI, and NR are excellent probes for the study of buried interfaces and larger-scale structures, a key region of interest for solid-state battery research. Better yet, some of the instruments possess both elastic and inelastic detectors, hence providing information about both the structure and dynamics in a single measurement. In situ and in operando experiments are a main focus of this review since this is a key strength of neutron scattering techniques that can unveil a new understanding of battery materials under operating conditions [9,10,13,31]. While our intent is to focus on solid-state batteries, some of the examples cover liquid electrolytes to demonstrate the unique information that can be obtained from neutron scattering techniques.

## 2. Structure

X-ray diffraction (XRD) is a leading structural characterization technique, but the low sensitivity to lighter nuclei and shallow penetration depth of photons can sometimes limit its applicability and effectiveness for battery systems. In these cases, atomic positions have to be inferred from the positions of the heavier atoms or by comparison to similar structures. Luckily, neutron diffraction can overcome these limitations and resolve these atomic positions. Another technique, known as total neutron scattering and PDF analysis, has been steadily gaining interest due to its ability to resolve both the global and local crystal structures simultaneously. Diffraction theory (neutron and X-ray) and pair distribution function analysis methods have been discussed in depth previously [34,35], along with instrument descriptions [36].

### Neutron Diffraction and Pair Distribution Function Analysis

Numerous studies have been conducted on battery materials using neutron diffraction techniques, most notably of which are in operando studies that are steadily becoming more common and routine for these instruments [37,38,39,40,41,42,43,44,45,46]. The long-range crystal structures are obtained by performing a Rietveld refinement to model the experimental neutron diffraction pattern. This allows for the determination of many structural parameters, such as the atomic positions, thermal displacement parameters, lattice parameters, site occupancy factors, preferred orientation, etc. [34]. In the diffraction pattern, coherent scattering produces Bragg peaks while incoherent scattering produces a broad background signal. Protonated samples should not be used for neutron diffraction experiments due to the large incoherent cross section of hydrogen. Rather, samples should be deuterated since the coherent cross section of deuterium is relatively high (5.592 barns), and the incoherent cross section is small (2.05 barns).

**Figure 1:**
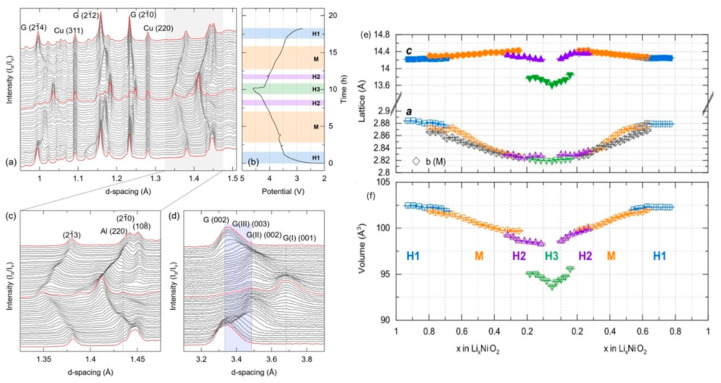
(**a**) Neutron diffraction patterns of a LiNiO_2_ | | graphite full cell during the first cycle [37]. (**b**) Voltage profile as a function of time. Evolution of the (**c**) LiNiO2 and (**d**) graphite Bragg peaks, (**e**) lattice parameters, and (**f**) unit cell volume during cycling as a function of Li concentration in Li_x_NiO_2_. Reprinted with permission from [37]. Copyright 2021 John Wiley and Sons.

In operando neutron diffraction experiments are able to observe changes in all the cell components simultaneously during charging and discharging. Chien et al. performed these measurements on a LiNiO_2_ | | graphite full cell [37]. As can be observed in Figure 1, the diffraction patterns evolve during the first charging cycle and show dramatic changes to the LiNiO_2_ cathode and the graphite anode. Bragg peaks corresponding to the Cu and Al current collectors can also be observed but do not change position during cycling, as expected. The LiNiO_2_ cathode undergoes a series of phase transitions, i.e., H1-M-H2-H3, where H and M represent hexagonal and monoclinic phases. Two-phase regions are also present while undergoing transitions between the different phases. Likewise, the graphite anode undergoes a series of structural evolutions from C-LiC_24_-LiC_12_-LiC_6_ upon Li intercalation. Following the first discharge, a splitting of the anode Bragg peaks is observed, corresponding to the unavoidable production of spinels (LiNi_2_O_4_ or Li_0.5_NiO_2_) and rock-salt-type (NiO) phases that are reversibly generated during this discharge process. As shown in Figure 1e, the *a*-lattice parameter for the Li_x_NiO_2_ cathode continuously contracts upon discharge. On the other hand, the *c*-axis increases in both the H1 and M phases before a decrease in the H2 phase. Upon further delithiation, a significant decrease in the *c*-axis is observed in the H3 phase. This is attributed to the collapse of the LiO_2_ interslabs due to a reduced charge concentration of the O atoms upon removal of Li^+^. The collapse of the layered structure causes a significant reduction of the unit cell volume, as shown in Figure 1f. Large volume changes in the electrodes during cycling are a major issue for solid-state batteries due to the difficulty in maintaining sufficient contact between the electrodes and electrolyte. To avoid this volume change, the upper cutoff voltage would need to be limited to avoid the formation of the H3 phase.

**Figure 2:**
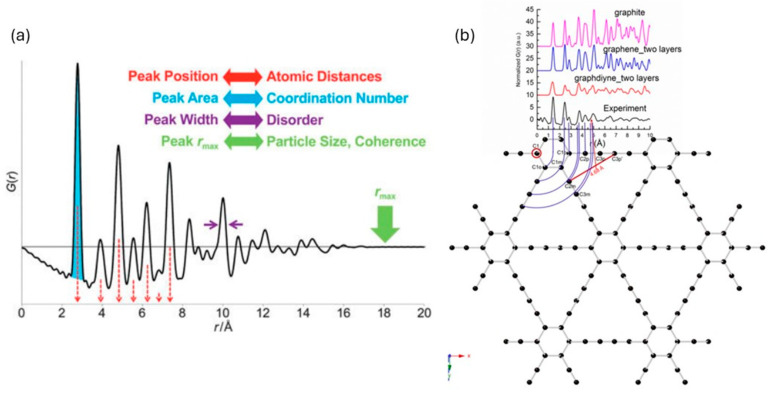
(**a**) Real space information that can be obtained from *G*(*r*) [47]. (**b**) Comparison of the atomic structure of graphdiyne to *G*(*r*) showing how the peaks are a real-space representation of atomic distances compared to an atom located at the origin [47,48]. Reprinted with permission from [47]. Copyright 2016 Springer Nature. Reprinted with permission from [48]. Copyright 2018 John Wiley and Sons.

In addition to Bragg scattering, there is a diffuse scattering signal located in between the Bragg peaks that is generally present as a weak signal with broad features. The diffuse signal contains a vast amount of information regarding the local structure and disorder. Unfortunately, the diffuse scattering signal is typically removed or modeled as a background during a Rietveld refinement. Hence, Rietveld refinements do not analyze the diffuse scattering signal and only probe the long-range, global structure. On the other hand, total neutron scattering experiments collect all the coherent scattering signal, which is produced by Bragg peaks (global structure), elastic diffuse scattering (static local structure), and inelastic diffuse scattering (atomic motion). By including the diffuse scattering in the analysis, total scattering experiments can reveal information about both the local and global structures simultaneously. The reduced pair distribution function (PDF) *G*(*r*) is obtained by performing a Fourier transform of the total scattering structure factor, S(Q), which is the normalized, measured total scattering intensity from the sample [35]. As demonstrated in Figure 2, *G*(*r*) is a real-space representation of how many atoms are located at a distance *r* from an atom located at the origin [47,48,49]. The peak position shows the atomic distance, while the peak area represents how many atoms share that coordination distance, which is weighted by their scattering power. The peak width shows the degree of disorder, with broader peaks representing more disorder. The general dampening of *G*(*r*) at long distances yields information about the coherent motion of atoms and the particle size. Figure 2b shows the structure of graphdiyne, a 2D carbon material with potential for use in high-performance electrodes [48]. Starting with an atom at the origin and moving radially outward, a series of coordination shells emerge that are directly connected to the peaks in *G*(*r*). By providing a real-space pair–pair coordination function, this powerful technique gives details about the atomic structure even for highly disordered or amorphous structures, where traditional Rietveld refinements provide little structural insight.

Similar to performing a Rietveld refinement to model diffraction data, PDF analysis is the method used for modeling total neutron scattering data. PDF analysis was originally developed to study the structure of liquids and amorphous materials, but its use has now been expanded to semi-crystalline and crystalline samples as well [35]. The interesting material properties are often governed by the defects or local structure. While the global structure may appear to be highly ordered, the local structure can be disordered, or vice versa, which can have profound effects on the material properties. Next-generation batteries may use amorphous or disordered electrodes to unlock enhanced performance [32,33].

**Figure 3:**
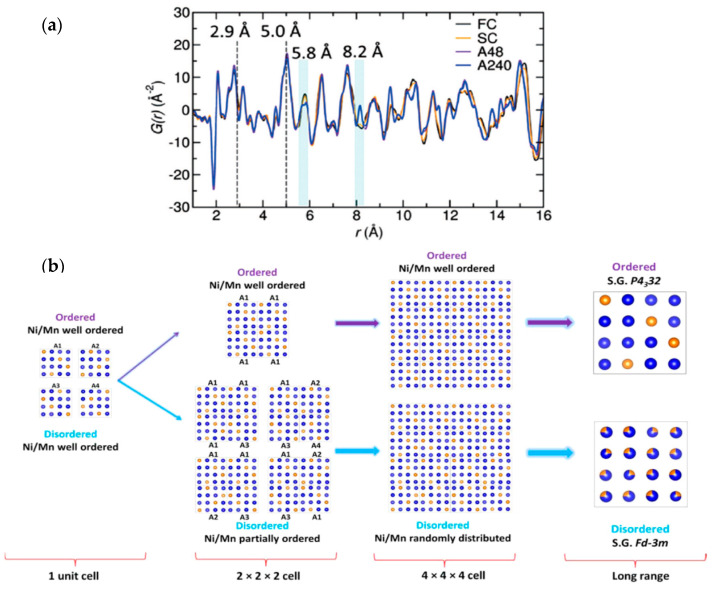
(**a**) *G*(*r*) for annealed (A48 and A 240) and non-annealed (FC and SC) LiNi_0.5_Mn_1.5_O_4_ samples [50]. The local structure is virtually identical for all 4 samples, but differences are observed at longer length scales corresponding to varying degrees of disorder. (**b**) Potential local vs. long-range ordering of the Ni/Mn sites to produce either an ordered or disordered global structure. Reprinted with permission from [50]. Copyright 2016 American Chemical Society.

Liu et al. conducted total neutron scattering experiments on four high-voltage spinel cathode samples (LiNi_0.5_Mn_1.5_O_4_) that were prepared using different heat treatment profiles: annealed (A48 and A 240) and nonannealed (FC and SC) [50]. The nonannealed disordered samples generally exhibit higher electrochemical performance. It was assumed that Ni/Mn were randomly distributed on 16d sites. The total neutron scattering experiment showed that the local structure corresponding to the length of one unit cell is virtually identical in each of the four samples. At longer length scales, differences in *G*(*r*) emerge corresponding to varying degrees of disorder/order, as shown in Figure 3. Locally, the Ni/Mn sites are well ordered. The authors found four plausible, well-ordered configurations (A1–A4) that can be derived using the space group P4_3_32, that is, the space group for the long-range ordered structure. As shown in Figure 3b, repeating units of the same configuration produce the ordered P4_3_32 structure, as expected. However, a superposition of different partially disordered configurations of type A1–A4 and of size 2 × 2 × 2 produces a disordered Fd-3m structure. Analyzing these configurations showed that Ni has a strong affinity to be coordinated to neighbor Mn sites, rather than Ni sites. Hence, this investigation shows that a short-range order is still maintained in the structure, but that larger domains composed of these subunits are disordered.

**Figure 4:**
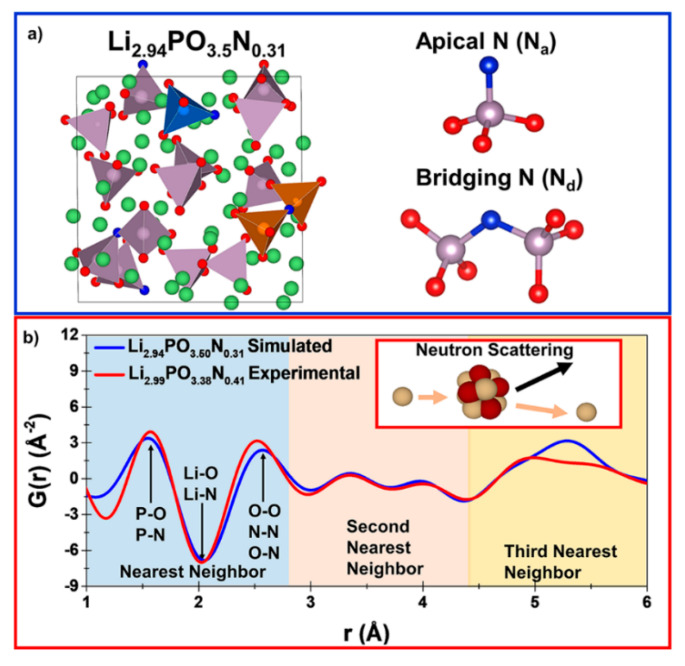
(**a**) Simulated crystal structure of Li_2.94_PO_3.5_N_0.31_ generated from ab initio molecular dynamics. O, N, Li, and P atoms are colored red, blue, green, and gray, respectively [54]. The apical and double-bridging N configurations are shown. (**b**) Comparison of the experimental and simulated PDF. Reprinted with permission from [54]. Copyright 2018 American Chemical Society.

Understanding the structure of amorphous materials has been a longstanding challenge that makes it difficult to pinpoint the structural origins of material properties. Some battery materials exhibit disordered or amorphous structures, such as shown in the X-ray PDF investigation by Key et al. of the crystalline–amorphous phase transition observed in silicon electrodes during (de)lithiation [51]. A promising amorphous solid-state electrolyte material is lithium phosphorus oxynitride, known as Lipon. By adding N to Li_3_PO_4_ to form Lipon, the ionic conductivity increases by more than an order of magnitude [52]. In addition, Lipon possesses increased electrochemical stability compared to Li_3_PO_4_ [53]. However, due to the amorphous structure, major inconsistencies existed between the different proposed atomic structures suggested from various experimental and theoretical methods [54]. Most of the uncertainty revolved around how the N atoms are incorporated into the structure. It was suggested that it is bonded to either two or three phosphate groups, known as double-bridging and triple-bridging. In addition, a third proposed bond was an unbridged apical bond with the phosphate group. A neutron PDF experiment by Lacivita et al. provided significant insight to finally resolve this structure [54]. The simulated structure from ab initio molecular dynamics is shown in Figure 4a, while the experimental and simulated PDF patterns are shown in Figure 4b. Overall, there is a good agreement between the experimental and simulated data for the local length scales up to 6 Å. The experimental and simulated sample stoichiometries are slightly different, which may be a reason why there are some noticeable differences in the experimental and theoretical PDF patterns. Multiple compositions of varying stoichiometry were tested in this work, and Li_2.94_PO_3.5_N_0.31_ best simulated the experimental PDF. It was found that as more Li and O were present in the sample, the peaks in the P–N partial PDF decreased while the P–O peaks increased. This means that adding Li reduces the amount of N that is double-bridged to two P atoms and instead increases the amount of apically bonded N. The P–P partial PDF also shows a dependence on the amount of double-bridged N because the bonding brings the P atoms closer together. Comparing the structural information to electrochemistry experiments shows that the presence of double-bridged N increased the ionic conductivity, but apical N does not. It is possible that when the bridged N pulls the two P atoms closer together, it opens pathways for the Li to diffuse through more efficiently.

## 3. In Situ/Operando Investigations of Interfaces and Bulk Components

Interfaces are one of the most critical regions of a battery. High-performance solid-state batteries require high-quality interfaces that are properly understood and optimized through successful interfacial design. These regions can be difficult to probe because they are inherently buried and cannot be easily accessed for measurements without disturbing the cell. However, these interfaces are generally formed dynamically and evolve during battery operation, so it is important to be able to monitor these processes in real time using in situ and in operando techniques [31]. The solid–electrolyte interface (SEI) is a crucial component of batteries that forms on the surface of the anode due to the electrochemical reduction of the solvent and salt species. This nanoscale layer protects the electrolyte solution from the highly reducing anode but also allows for the diffusion of ions through its structure. A stable SEI layer is necessary to prevent the degradation of active cell components and to limit capacity loss. The SEI is formed through multiple reaction routes consisting of chemical and electrochemical reactions, which results in a complicated and complex structure and chemistry. Many investigations of the SEI are conducted through ex situ measurements, which are routinely performed by cycling the battery and then removing the solvent through washing. However, washing the sample changes the chemical makeup and structure of the SEI by removing soluble species and retaining only the insoluble species, which necessitates the use of in situ/operando methods. Three neutron scattering techniques that can be used to measure the larger length scales applicable to the SEI, interfaces, and bulk components are small-angle neutron scattering (SANS) [55], neutron reflectometry (NR) [56], and neutron imaging (NI) [57,58].

### 3.1. Small-Angle Neutron Scattering

SANS is a technique used to investigate large-scale structures by measuring the scattered signal at small angles. The momentum transfer, *Q*, scales as a function of half the scattering angle, *θ*, and incident wavelength, *λ*, as *Q* = (4*π* sin *θ*)/*λ*. With *Q* being inversely proportional to the real-space length scale, *d*, through *Q* = 2*π*/*d*, large structures therefore scatter neutrons at small scattering angles. Multiple investigations have employed SANS to investigate the structure and chemistry of battery materials [59,60,61,62,63,64,65]. Sacci et al. used it to investigate the SEI formation on a graphite electrode using an ethylene carbonate/dimethyl carbonate (EC/DMC) electrolyte solution [62]. In order to simplify the chemistry and structure of the SEI, some potential reactions were intentionally avoided by not including any lithiated salts in the electrolyte. Instead, the carbon anode was prelithiated to Li_6_ or Li_12_ before being reacted with the EC/DMC solvent to form the SEI. To further simplify the picture, the cell was not cycled to avoid electrochemical reactions, such that the only reaction that should take place is the chemical reaction of the lithiated carbon with the solvent.

The scattering length density (SLD), *ρ*, of a material is a summation of the coherent scattering length over all the atoms in a given unit volume. The scattered intensity, *I(Q)*, for a two-phase system is proportional to the square of the difference in the SLD for the two phases, (Δ*ρ*)^2^. Carbon has a large SLD, while voids have a SLD of zero. Therefore, voids in carbon scatter very strongly. Li has a negative SLD, which means that Li intercalation of the graphite will reduce the scattering intensity compared to the voids. When the EC/DMC solution fills the voids, it also reduces the scattering intensity. In addition, isotope substitution can be used to vary the SLD to separate the contributions in complex systems, a technique known as contrast variation. For example, hydrogen can be replaced by deuterium in the EC/DMC solution to identify which parts of the system are hydrogenated, such as the polymeric SEI layer. In this case, deuterating the solvent leads to a further reduction in the scattering intensity. Due to the SLD contrast at the interfaces, the carbon–SEI interface is better studied using the protonated solvent, while the deuterated solvent is better for studying the void–SEI interface.

**Figure 5:**
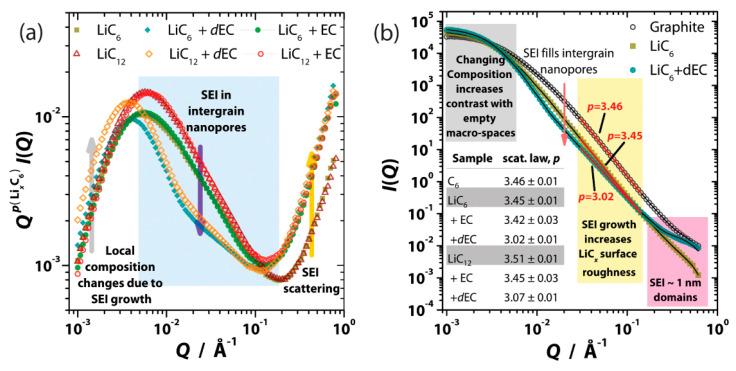
(**a**) SANS curves normalized by *Q^p^* for the pristine LiC_x_-air system to highlight changes in the intergrain nanopores [62]. (**b**) SANS curves for graphite, LiC_6_. And LiC_6_ + dEC. Inset has the scattering law exponents, showing that the SEI roughens the graphite surfaces. Reprinted with permission from [62]. Copyright 2015 American Chemical Society.

As shown in Figure 5b, the SANS signal decays as *I(Q)* ∝ *Q*^−*p*^ in the *Q*-range of 0.02 to 0.1 Å^−1^ [62]. The scattering exponents for graphite and LiC_6_ are *p* = 3.46 and 3.45, respectively. Hence, the graphite surface creates fractal scattering, indicating a rough surface. After the SEI coats the surface, the scattering exponent decreases to *p* = 3.02, which is very close to mass-fractal-type scattering (*p* < 3). Therefore, the SEI layer coats the carbon surface with an even rougher structure. In Figure 5a, the data are normalized by *Q^p^* for the pristine LiC_x_-air mixture to better highlight changes in the intergrain pores. There is a slight increase in the intermediate *Q*-range (0.005–0.1 Å^−1^) following a reaction with the protonated solvent, and a larger decrease is observed using the deuterated solvent, hence showing that the SEI layer is hydrogenated. Above *Q* = 0.2 Å^−1^, the scattering increases for both the protonated and deuterated solvents due to the formation of nanodomains along the SEI/Li_x_C_6_ interface. A reduction of DMC would produce Li_2_CO_3_, while a reduction of EC would produce a polymeric layer. A drastic difference in scattering intensity is observed using contrast variation with SANS (along with INS results that are discussed later in the INS section), which further supports these studies that the SEI is polymeric in nature. Hence, the reduction of EC is preferred over the reduction of DMC.

The SEI is critical for the safety and performance of batteries. It has been shown that higher concentrations of electrolyte can produce a more stable SEI layer as compared to the traditional, more dilute solutions [66,67]. The in operando SANS experiment by Jafta et al. examined how the electrolyte concentration changes the surface chemistry and microstructure of the SEI [64]. The cell used an ordered mesoporous hard carbon electrode and 1 M LiTFSi/PC and 4 M LiTFSi/PC electrolyte solutions (LiTFSi = LiN(SO_2_CF_3_)_2_, PC = propylene carbonate). Following 100 cycles, the capacity of the 4 M cell remains relatively constant while the 1 M cell experiences a gradual decrease in capacity. The 1 M cell has a higher relative capacity due to the higher degree of intercalation. The higher viscosity of the 4 M cell limits diffusion and decreases the relative capacity, but the SEI layer exhibits more stable cycling behavior. SANS and X-ray photoelectron spectroscopy were then employed to compare the differences in performance to changes in the microstructure of the SEI layer. There is a vast amount of information revealed from these measurements, of which we will just highlight the main findings.

**Figure 6:**
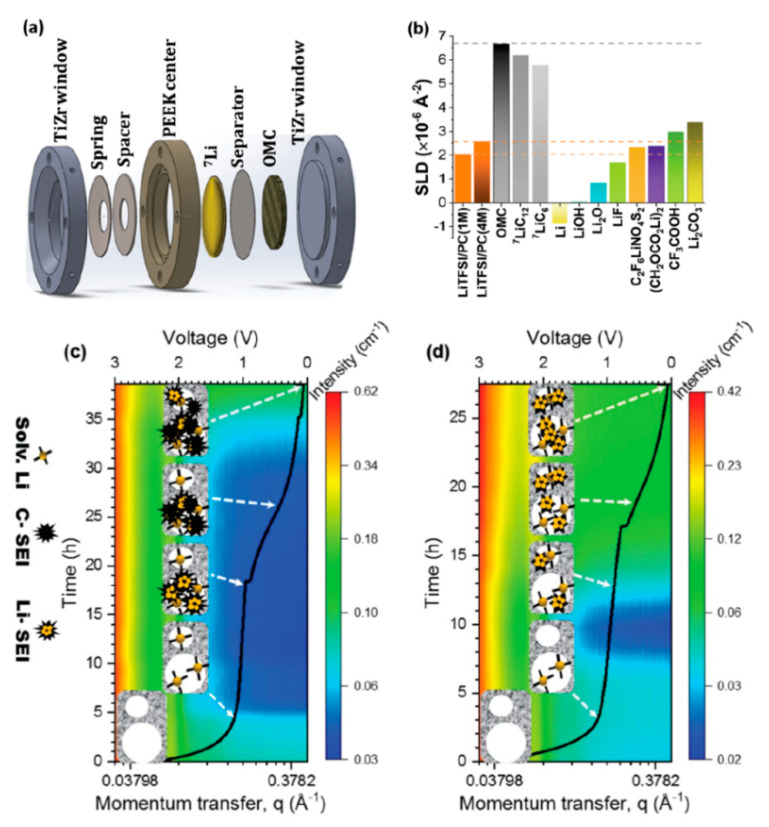
(**a**) Schematic view of the cell used for in operando SANS measurements [64]. (**b**) Scattering length densities for the cell components and major SEI components (electrolyte reduction products). Dashed lines represent the contrast difference between the two electrolytes. SANS intensity as a function of first discharge time, momentum transfer, and cell voltage for (**c**) 1 M LiTFSi/PC and (**d**) 4 M LiTFSi/PC electrolyte solutions. Reprinted with permission from [64]. Copyright 2019 RSC.

The scattering intensity is shown in Figure 6c,d as a function of first discharge time, momentum transfer, and cell voltage for the 1 M and 4 M samples, respectively [64]. There are clear differences observed in the scattering intensity trends for both samples. Once again, the decay of the scattering intensity at low *Q* can be used to investigate the surface roughness, whereas the intensity at higher *Q* yields information about the micropores and their surrounding environment. First, the formation of the electric double layer (EDL) is observed from the open circuit voltage (OVC) to 1.1 V as the solvated Li^+^ are adsorbed onto unoccupied micropore and mesopore walls. For the 1 M sample, the scattering intensity corresponds to the SLD of Li^+^(PC)_4_-filled mesopores, while the 4 M sample corresponds to a solvation shell consisting of TFSI^−^ and Li^+^(PC)-TFSI aggregates filling the mesopores. In the micropores, a decrease is observed in the scattering intensity for the 1 M sample according to solvated Li^+^ adsorption, but the 4 M sample intensity remains constant. This suggests that the higher viscosity prevents adsorption of the 4 M electrolyte in the micropores, which does not start to fill up until around 1 V. In the 1.1 to 0.9 V range, co-intercalation of partially and fully solvated Li^+^ begins to occur in the graphitic layers. The solvent also begins to reduce in the mesopores but not in the micropores. The reduction of LiTFSi forms a Li-rich SEI layer, while the decomposition of PC forms carbonaceous compounds. For the 4 M sample, LiTFSi begins to decompose in the mesopores at a lower voltage, but there are fewer carbonaceous compounds compared to the 1 M sample. Over the voltage range of 0.9 to 0.3 V, the composition of the micropores begins to fill up with Li-rich compounds in the 4 M sample. This occurs at a much lower potential in the 1 M sample (0.3 to 0.05 V).

After the cell is fully discharged, there are key differences in the resulting SEI layer depending on the electrolyte concentration. While the mesopores are filled with both Li and carbonaceous components for the 1 M sample, the scattering becomes dominated by the carbonaceous species once the cell is fully discharged. On the other hand, the 4 M sample is dominated by Li-rich compounds (Li salts), while the signal from carbonaceous compounds is small. The micropores for both samples are filled with Li-rich compounds. The SEI layer that ultimately forms is thicker for the 1 M sample compared to the 4 M sample, likely due to the increased amount of carbonaceous compounds due to increased PC reduction. As this study shows, SANS can be an extremely powerful technique to probe changes in the SEI microstructure in truly in operando experiments, much of which information cannot be obtained by other methods.

### 3.2. Neutron Reflectometry

Neutron reflectometry probes the surfaces and interfaces of thin films by measuring the specular and off-specular reflectivity of neutrons from the surfaces of materials. Similar to SANS, neutron reflectometry can provide information about the structure, chemical makeup, and formation of the SEI in truly in situ measurements [66,68,69,70,71,72,73,74,75,76].

**Figure 7:**
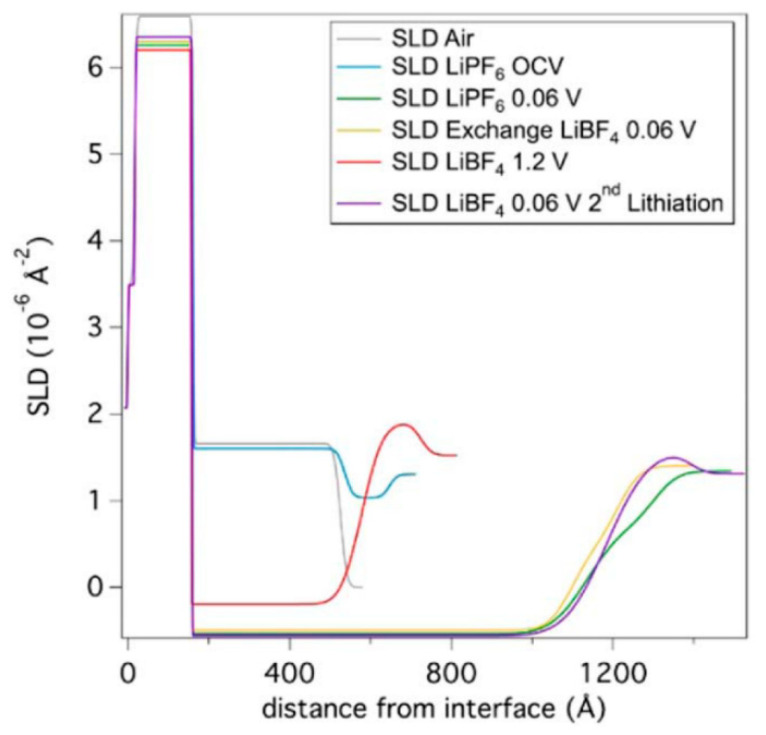
Scattering length density as a function of distance from the substrate [72]. Reprinted with permission from [72]. Copyright 2021 IOP.

In an interesting study, Veith et al. demonstrated how post-cycling electrolyte replacement can provide additional insight into the SEI formation [72]. The cell consists of a 5 mm thick Si substrate, an amorphous Si working electrode, the SEI layer, and the electrolyte. The electrolyte is a standard LiPF_6_ salt, EC/EMC solution (EC = ethylene carbonate, EMC = ethyl methyl carbonate). Figure 7 shows the scattering length density as a function of distance from the substrate. The amorphous Si and SEI thicknesses are listed in Table 1 [72]. Initially, the amorphous Si layer is 365 Å thick. The OCV corresponding to a lithium-free Si anode and lithium metal is 2.7 V. Once the cell is assembled, an initial formation of the SEI takes place in less than an hour, is 110 Å thick, and corresponds to the segregation of lithium ions on the surface of the electrode. Upon discharge to 0.06 V, the lithium intercalates the anode and increases the Si thickness to 976 Å, as observed in Table 1. The SLD of this layer decreases drastically due to the incorporation of lithium with an estimated stoichiometry of Li_15_Si_4_. The SEI also expands to 176 Å.

At this point, the electrolyte was removed and replaced with a LiBF_4_ EC/EMC electrolyte solution. A subsequent measurement shows that the thickness of the SEI layer has been reduced by roughly a third to 115 Å, indicating that a significant portion of the SEI is easily removable. Comparing the SEI that formed during the OVC measurement to the SEI formed following solvent exchange, there are clear chemical differences. This highlights the importance of performing in situ measurements, as compared to washing the electrode and studying the SEI layer ex situ. In this case, washing likely removed weakly bound P–F salt species and soluble organic molecules. Furthermore, it was found that the SEI is a rough or diffuse layer with little porosity and that there were no signs of layered heterostructures. Upon further cycling, the SEI layer expands, which is caused by the decomposition of solvent molecules and the deposition of the products in the SEI. These results suggest that the easily removable salt species play a key role in the degradation rate of organic solvents.

**Figure 8:**
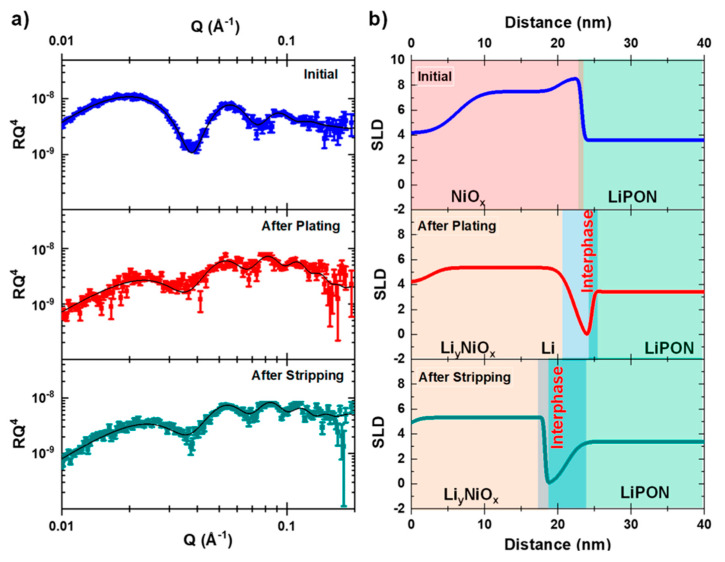
(**a**) Neutron reflectometry data for a NiO/LiPON solid-state battery for the (blue) initial pristine state, (red) after Li plating, and (cyan) after Li stripping [73]. The data are plotted as *RQ*^4^ to enhance the profile features. The black lines are a fit to a multi-layer thin film structure. (**b**) Scattering length density profiles (SLD) calculated from fits to the reflectivity data with a diagram showing the thickness of the different layers. Reprinted with permission from [73]. Copyright 2023 American Chemical Society.

Neutron reflectometry has been used extensively to probe liquid electrolytes and the SEI [66,74,77,78]. Recently, it has been extended to examine the solid–solid interface between Li metal and Lipon by Browning et al. [73]. The sample was deposited on a quartz substrate and consisted of a 20 nm thick layer of NiO (was intended to be Ni), followed by ≈1 μm of Lipon, and lastly a 10 μm Li metal layer. This investigation focused on the NiO/Lipon interface and conducted a series of plating and stripping processes by passing Li through the Lipon to the NiO surface to form the interface. Figure 8 shows the reflectometry data, *R*, plotted as *RQ*^4^ vs. *Q* to enhance the higher-*Q* features in the profile. The black lines are a fit to a multilayer thin film model with values guided by electrochemical measurements. NR measurements of the initial (unplated) stack show two distinct layers, namely an O-rich layer near the quartz substrate and a Ni-rich layer near the Lipon surface. After Li plating, the NiO layer expands from 17 to 20 nm, and the SLD decreases, suggesting the formation of a Li_x_NiO_y_ compound. In addition, a Li layer is observed to form that is found to be 2.4 ± 0.4 nm thick and has a SLD that is close to the value for Li metal. Also, both the NiO/Li and Li/Lipon interfaces have substantial roughness at 12.0 Å and 6.5 Å, respectively. The physical meaning of surface roughness values from this type of measurement can be difficult to interpret, but in this case, it may relate to a physical roughness (inhomogeneity between the layers) or perhaps from a chemical gradient. Following the stripping process, a distinct Li-rich interphase remains, which requires being fit with a structural model containing both a residual Li metal phase and a Li-rich phase. It is likely that this phase is not a stoichiometric phase (e.g., Li_2_O, Li_3_P, or Li_3_N) and could also be amorphous. The SLD for this phase also increased, which suggests that some of the Li has been removed during the stripping process. Interestingly, the roughness for the NiO/Li-rich layer decreases to 2 Å, but the opposite occurs for the Li-rich layer/Lipon interface, which increases to 15.7 Å. Considering the roughness, the thickness of this interface is estimated to be around 6–7 nm thick. Therefore, this experiment demonstrated that solid–solid battery interfaces can be dynamically formed in situ and non-destructively probed with nanometer precision using neutron reflectometry techniques.

### 3.3. Neutron Imaging

Neutron radiography and computed tomography (CT), collectively referred to as neutron imaging, are powerful techniques used to image the internal features of materials. In-depth overviews of neutron imaging and advanced imaging techniques have been covered elsewhere [57,58], including some recent reviews on its application to battery materials [28,29,30]. Similar to X-ray imaging and CT scans used in medical and industrial applications, the incident beam is attenuated by the different components in an object and can be used to form an image. However, the scattering potential of neutrons allows for a unique contrast between elements that may be challenging to observe using X-rays. Luckily for Li-based batteries, the absorption cross section for ^6^Li is very large (940 barns), which causes a sharp contrast between Li and other cell components, allowing for the Li positions to be readily resolved. In a basic neutron imaging experiment, a scintillation detector is located behind the sample such that neutrons that are transmitted through the sample interact with the detector and are converted to light. This light is then measured by a digital detector and converted into a 2D image (radiograph). The sample can be mounted on a rotation stage such that a series of measurements at different orientations can be computationally combined to form a 3D image (computed tomography).

Numerous neutron imaging and CT experiments have been conducted on battery systems to investigate Li distribution and transport [79,80,81,82,83,84,85,86,87], volume swelling [85,88], gas evolution [89,90], cell failure due to dendrite formation [86], and electrolyte studies [91,92]. Interfacial boundaries are a key region of focus for battery imaging research. As battery components swell and shrink during cycling, it can be challenging to maintain adequate contact between solid-state battery components. Expansion and contraction of solid materials can also result in cracking and other structural defects. These defects can be readily studied non-destructively using complementary X-ray and neutron imaging techniques. This section will cover neutron imaging studies for examining dendrite formation and cell failure mechanisms, investigation of cracks and delamination, lithium transport and spatial distributions, and virtual unfolding and slicing of battery components using advanced data reduction methods.

**Figure 9:**
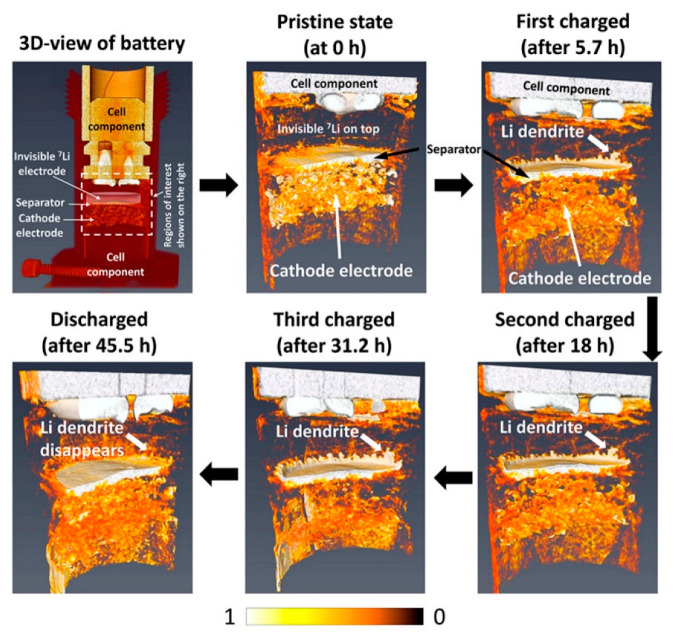
Evolution of the Li distribution in the battery at a series of charge/discharge stages. High attenuation due to the presence of ^6^Li is colored white. Dendrite formation is observed during charging and disappears upon discharge [86]. Reprinted with permission from [86]. Copyright 2019 American Chemical Society.

Rechargeable batteries using Li metal anodes are considered the “Holy Grail” of energy storage systems due to the very high theoretical energy capacity and the lowest negative electrochemical potential [19,86]. However, uncontrollable dendrite formation is a major issue that hampers its use in practical applications. To better understand the dendrite formation process, Song et al. used operando neutron imaging on a Li metal anode battery [86]. A schematic of the battery is shown in Figure 9, along with a series of 3D rendered volumes that were reconstructed from the neutron tomography data. The half-cell used a ^7^Li anode, which is essentially invisible to neutrons, but the active component, LiMn_2_O_4_, used the highly absorbing natural lithium so that the dendrite growth and Li distributions could be observed. The electrolyte was deuterated (d-ethylene carbonate and d-dimethyl carbonate) to reduce the scattering potential but contained LiPF_6_ salt consisting of natural lithium that is highly visible. A glass fiber cloth soaked in electrolyte surrounds the inner wall of the cell to prevent internal shorting and provides the least resistant diffusion pathway for Li^+^. Upon charging, large branch-like dendrites can be observed forming along the glass cloth and continue to grow as the cell charges. The Li distribution in the cathode also changes significantly as the Li is removed during charging. Upon discharge, the Li dendrites disappear.

Following cell failure, neutron imaging shows that the short circuit causes areas on the outside of the cathode to form a region with two distinct parts: a highly absorbing inner region and a highly transmissible outer region. This is due to the thermal deformation of the cathode, which causes a void space to form between the cathode and cell wall. Rather than having Li^+^ transport along the glass fiber, the Li now collects on the edge of the cathode. When the cell shorts, a self-discharge process occurs between the dendrite and cathode as it forms a low-resistance pathway for electrons to travel from the dendrite into the cathode, where it reduces Li^+^ species to form LiMn_2_O_4_. As the dendritic Li is removed and LiMn_2_O_4_ is formed, the dendrite shrinks and ultimately causes a gap to form between the dendrite and cathode. Due to the lack of physical contact, the electronic transportation pathway is broken, the self-discharge process is halted, and the cell begins charging again. Subsequently, the dendrite grows, and a short circuit occurs again. This competitive process repeats continuously in a cycle. An interesting video of the time evolution of the dendrite growth and cell failure during charging is included in the Supplementary Information section of the work by Song et al. [86]. This investigation shows how in operando neutron imaging and computed tomography can provide powerful insight into the time-dependent Li distribution and ionic transport, as well as dendrite formation and cell failure mechanism.

**Figure 10:**
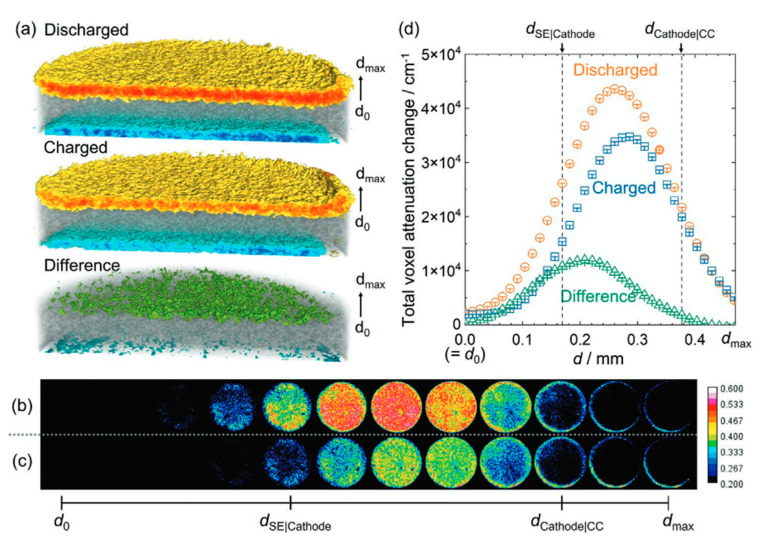
Neutron tomography for solid-state sulfur cathodes. (**a**) 3D reconstruction of the discharged (Li in cathode) and charged states, along with the difference showing the location of the mobile Li shown in green. Neutron imaging slices of 39 μm thick slices of the cathode showing the lithium distribution for the (**b**) discharged and (**c**) charged states [93]. Reprinted with permission from [93]. Copyright 2023 John Wiley and Sons. (**d**) Total attenuation change for the discharged (orange) and recharged (blue) states. The difference (green) shows the mobile Li.

Bradbury et al. conducted neutron tomography to investigate the distribution of lithium as a function of depth in solid-state sulfur cathodes [93]. This study uses a lithium-thiophosphate-based solid-state Li–S battery consisting of In/Li|Li_6_PS_5_Cl|S/C/Li_6_PS_5_Cl. A 3D tomograph is shown in Figure 10a for the discharged and charged states. Following discharge, the Li transfers from the cathode to the anode, which causes the anode to show a negative attenuation change, the cathode a positive attenuation change, and the separator region has zero net change in total lithium, as denoted in gray. Li mobility and distribution can be visualized as a function of depth by examining the difference in attenuation for the two charge states in Figure 10d, as well as a series of 39 μm thick slices of the cathode in Figure 10b,c. This shows that the mobile Li is located on the anode/separator side of the cathode (shallower depth), while immobile Li that is trapped in the cathode is located in greater amounts on the current collector side of the cathode (deeper depths). The orange-colored slices show the heterogeneous lithium distribution in the cathode following discharging to form Li_2_S, while the recharged state shows the leftover lithium that is trapped in the cathode.

**Figure 11:**
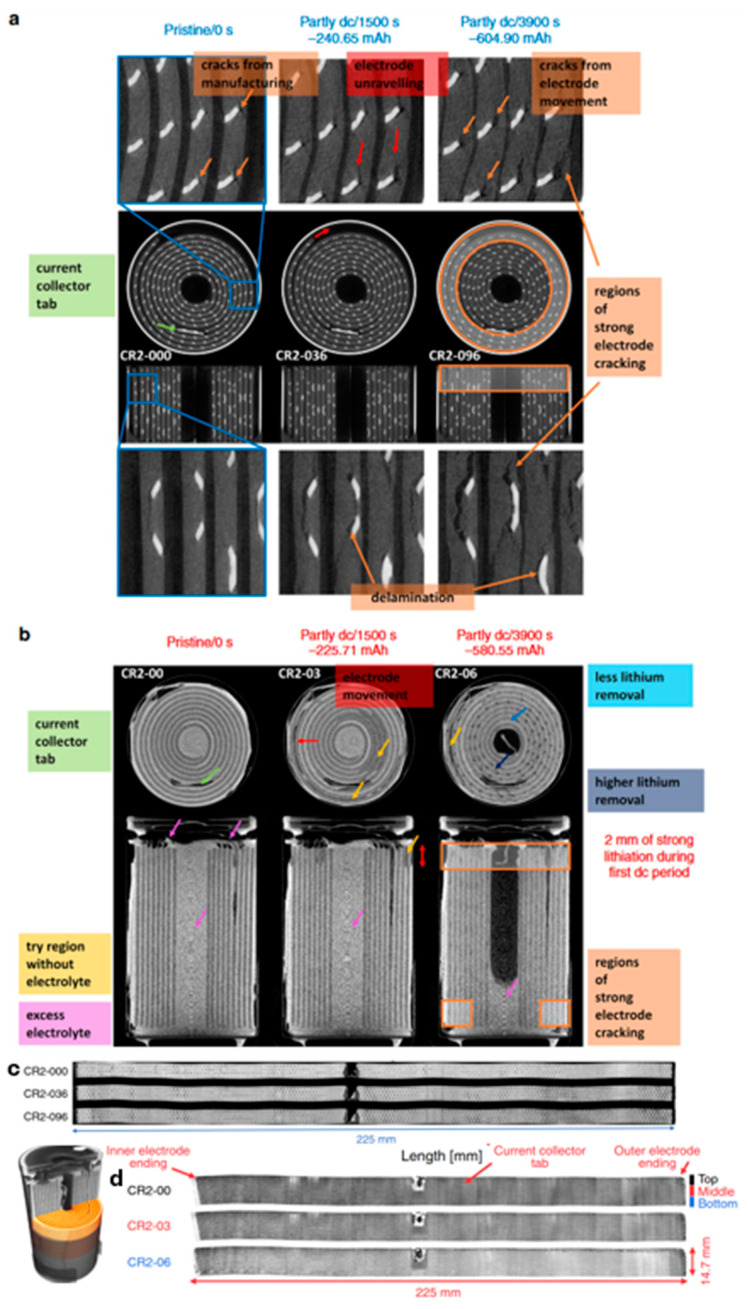
(**a**) X-ray and (**b**) neutron imaging of commercial Li/MnO_2_ cells that contain a wound electrode/separator/current collector assembly. The X-ray images are sensitive to the highly attenuating Ni current collectors, while the neutron technique is sensitive to the Li distributions and electrolyte. Cracks and delamination can be observed by X-rays in the cathode as the cell expands upon lithium insertion. Different colored arrows highlight regions of interest with features explained in the correspondingly colored text boxes. Virtual unfolding of the separator/electrode assemblies using (**c**) X-rays and (**d**) neutrons [88]. Reprinted with permission from [88]. Copyright 2020 Springer Nature.

Imaging studies that combine both neutron and X-ray experiments are a powerful approach to unlock even greater detail about the battery. The following work by Ziesche et al. presents an interesting example of how thin, spiral-wound electrode layers in a cylindrical battery cell can be virtually unwrapped using advanced data reduction techniques [88]. This study used commercial batteries (CR2 Li/MnO_2_) that contained a wound electrode/separator assembly with a Ni current-collecting mesh woven into the cathode. X-ray imaging is uniquely suited for detecting mechanical degradation processes, such as cracking, delamination, or unravelling effects. It is also higher resolution and can be leveraged to identify features that are beyond the resolution limit of neutron CT. However, X-rays are not able to observe the electrolyte, or the Li (de)intercalation process, and therefore must be inferred from volume changes. The pristine state of these batteries contained cracks from the manufacturing process that grew heterogeneously upon cycling as the components underwent volume changes. The delamination observed around the current collectors can create electrically (electrochemically) isolated areas, which can cause power and capacity losses. However, cracks that expose additional Li_x_MnO_2_ surface area (porosity) could benefit the Li insertion process. Neutron imaging is more sensitive to probing the Li distributions as well as tracking electrolyte consumption. Electrolyte consumption can be observed in Figure 11, where the middle region of the battery is initially full of electrolyte (bright) and is consumed gradually during discharge until the inner axial region appears dark. To explore the evolution of electrode assembly as a function of length in the spiral-wound layers, the tomograms were virtually unrolled using data reduction methods, as shown in Figure 11c,d. These cut-out sections of the cathode are for different states of charge to show how the electrode is lithiated differently depending on its position in the battery. For example, differences in compression amongst the wound–electrode layers caused the upper and outer axial sections to experience more pronounced cracking and delamination. The neutron data from these regions showed very high activity, with a preference for Li intercalation in the outermost layers due to the lower compression and better wetting by the electrolyte.

## 4. Dynamics

While neutron diffraction measures elastic scattering (no energy transfer) for structural investigations, neutron spectroscopy measures the energy (*ħω*) and momentum (*ħQ*) transferred in the scattering process, known as inelastic scattering. Small energy transfers (µeV to a few meV) are observed as a Doppler-like broadening of the elastic peak. This broadening arises, for instance, from reorientational or translational diffusion, and it is called quasielastic neutron scattering (QENS). Energy transfers larger than a few meV can be measured using inelastic neutron scattering methods to study phonons and molecular vibrations. Neutron spectroscopy theory has been discussed previously [94,95,96]. While battery experiments are becoming fairly routine for certain neutron techniques such as diffraction, SANS, reflectometry, and imaging, we feel that neutron spectroscopy as a whole remains underutilized in this field, apart from the routine use of QENS for ionic transport studies. We hope that this section can help spur growth and interest for neutron spectroscopic studies of battery materials.

### 4.1. Inelastic Neutron Scattering

Inelastic neutron scattering (INS) is an analytical technique that measures the vibrational density of states over a wide energy transfer range [95,97,98]. This provides information about chemical bonding, atomic structure, intermolecular vibrations, and chemical reactions. A neutron vibrational spectrometer is the neutron equivalent of a Raman spectrometer. While certain vibrational modes are not active in light scattering techniques due to selection rules, there are no selection rules for INS, and hence all modes are observable. Numerous studies have used inelastic neutron scattering to probe the vibrational dynamics of battery materials, of which we will discuss some examples in detail below [99,100,101,102].

**Figure 12:**
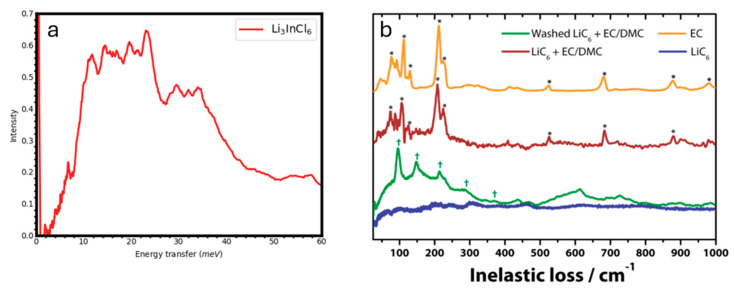
(**a**) INS spectra of Li_3_InCl_6_ measured at 5 K at the VISION spectrometer [100]. (**b**) INS spectra for EC, LiC_6_, LiC_6_ + EC/DMC, and washed LiC_6_ + EC/DMC [62]. The asterisks (*) correspond to EC peaks, while daggers (†) are associated with PEO-type peaks. Reprinted with permission from [62]. Copyright 2015 American Chemical Society.

Li_3_InCl_6_ is interesting as it is an air-stable battery electrolyte with high conductivity, ~10^−3^ S.cm^−1^. The material is monoclinic and has eight non-equivalent Li sites. Li and In atoms are surrounded by six Cl atoms in octahedral coordination. Li and In octahedra can share corners or edges to form a complex unit cell with 120 atoms. The librations of the octahedra play a role in the diffusion of Li cations through the structure. The neutron vibrational spectrometer VISION at Oak Ridge National Laboratory offers a dynamic range of 0–8000 cm^−1^ with a resolution of ~1–1.5% across this range [103]. Its use of neutrons for vibrational spectroscopy has the advantage over optical spectroscopies that an average is obtained over the entire Brillouin zone. In particular, zone-boundary phonons, which play a role in various physical phenomena (e.g., phase transitions), are observable. Figure 12a shows the phonon density of states of Li_3_InCl_6_ measured at 5 K [100]. The low frequency range of the spectrum shows the classic Debye behavior (ω^2^). A large number of phonons are resolved. This phonon density of states can be calculated quantitatively, for example, with DFT or AIMD techniques, and compared directly to the experiment to associate the vibrational mode (frequency and atom displacements) to the various features in the experimental spectrum. Variations in vibrational mode position or intensity with various physical parameters, such as temperature or applied AC electric field, are useful to identify the type of motions (i.e., phonons) involved in ionic transport.

Vibrational spectroscopy can also be applied to investigate the chemical composition of the SEI layer, as demonstrated by Sacci et al. in the same study that we discussed earlier in the SANS section [62]. Figure 12b shows a series of INS spectra corresponding to EC, LiC_6_, LiC_6_ + EC/DMC, and washed LiC_6_ + EC/DMC. The washed sample was rinsed with DMC three times and dried for 24 h. Unsuccessful attempts were made to obtain vibrational spectra using infrared and Raman spectroscopy because the spectra were dominated by the graphite signal. On the other hand, neutron vibrational spectrometers are highly sensitive to hydrogen. Despite using around 2 g of LiC_6_ sample, the carbon does not contribute much intensity to the INS spectra. Rather, the spectra are dominated by the nanoscale-sized hydrogenated SEI layer. The LiC_6_ + EC/DMC spectrum mostly consists of the EC signal. After the sample is washed, the EC signal is mostly removed, which allows for an observation of the SEI layer. The peaks in this signal resemble poly(ethylene oxide)-type (PEO), which suggests that the SEI layer is polymeric in nature. The SEI layer observed in this measurement was created chemically by using pre-lithiated graphite and reacting it with EC/DMC electrolyte solution. Hence, electrochemical reactions were avoided, and it shows that the chemical reaction alone creates a simpler, polymeric, PEO-type SEI structure. It is expected that reduction of DMC will produce Li_2_CO_3_ while reduction of EC produces Li_2_(CH_2_CH_2_CO_2_)_2_. Therefore, Li reduction of EC is preferred over DMC.

Since diffusion is inherently a thermally activated process, it is important to understand the lattice dynamics. An investigation by Muy et al. used INS and computational modeling to better understand how to tune the lattice dynamics of solid-state Li conductors to increase the stability of the electrolyte against the cathode while maintaining fast ionic transport [99]. Undesirable reactions between Li-based solid-state electrolytes and the cathode are a major issue for battery research. Likewise, materials that have low anion phonon centers experience a reduction in stability due to oxidation at the cathode. On the other hand, fast Li conduction is observed in materials with low Li phonon mode centers. Therefore, materials that possess both low Li band centers and high anion centers are promising candidates for fast ionic conductors with increased stability. Ionic mobility can be related to the phonon modes through the assumption that larger deviations from the atomic sites will allow the ion to explore neighboring sites, hence leading to more efficient transport. Such excursions will occur in soft atomic potentials located at lower phonon mode energies. Therefore, it is desirable to have lower energy Li modes for increased transport properties.

**Figure 13:**
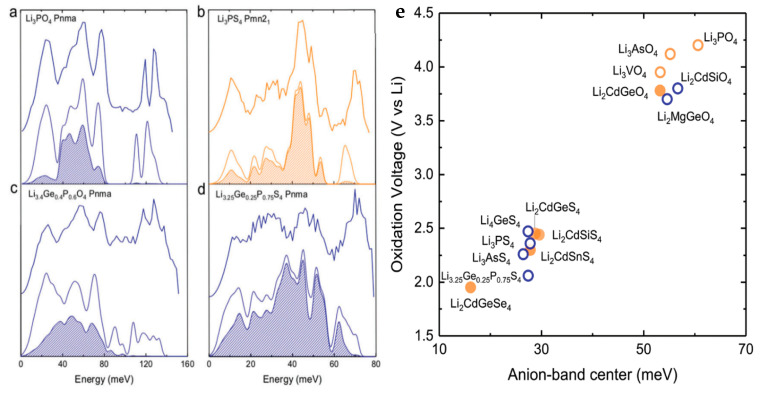
INS phonon density of states for (**a**) Li_3_PO_4_, (**b**) Li_3_PS_4_, (**c**) Li_3.4_Ge_0.4_P_0.6_O_4_, and (**d**) Li_3.25_Ge_0.25_P_0.75_S_4_. The top line are the experimental data collected at 100 K, the bottom line is calculated computationally at 0 K, and the shaded region is the Li phonon contributions. (**e**) A comparison of the oxidation voltage as a function of anion phonon band center [99]. Reprinted with permission from [99]. Copyright 2018 RSC.

In this investigation, numerous lithium superionic conductors (LISICON) based on Li_3_PO_4_ and olivine samples were investigated experimentally and computationally. Systematic changes were induced in these compounds through cationic and anionic substitution to tune the lattice dynamics. Experimental INS measurements are shown in the top lines in Figure 13a–d, while the lower lines are the computational calculation. In addition, the Li contributions to the spectra are highlighted by the shaded pattern. Substitution of S for O leads to increased softening of the Li modes. For example, Li_3_PO_4_ has lithium modes at energies up to 80 meV, but by substituting S for O, the modes for Li_3_PS_4_ have now been softened to energies below 60 meV. Likewise, this substitution results in a reduction of the activation energy from 1.4 eV [104] to 0.5 eV [105] and a reduction in the migration barrier from approximately 0.7 eV to 0.3 eV, hence indicating improved ionic mobility [99]. As mentioned previously, a softening of the anion band centers is associated with reduced stability against oxidation. The oxidation potential is defined as the voltage above which the material will be oxidized following the removal of Li^+^ from the lattice. Figure 13e shows a comparison of the oxidation potential as a function of anion band center energy. A clear relationship is present in which a lower anion band reduces the oxidation potential. While substituting O with S resulted in enhanced lithium mobility, it also caused a significant decrease in the oxidation potential, i.e., a reduction of around 30 meV caused a drop in the oxidation potential from approximately 4 V to 2.5 V vs. Li. The authors attribute the reduction in stability to an increase in anionic mobility, which affects the kinetics and thermodynamics to promote reactions of the solid-state electrolyte with the electrode. Unfortunately, a trade-off between Li mobility and stability occurs in these materials. Nonetheless, INS provided key insight into how the lattice dynamics directly affect these properties. With this information, efforts can now be used to look for materials that would be better suited as solid-state electrolytes and provide guidance for better tailoring the properties of existing electrolytes.

### 4.2. Quasielastic Neutron Scattering

QENS instruments are ideally suited for investigating the ionic transport and single-particle dynamics in solid-state battery materials. This technique can reveal atomic-scale diffusion pathways and mechanisms that are vital to unlocking the fast ionic conduction required for high-performance devices and fast charging. Comprehensive details about QENS scattering theory can be found in the following books [94,106], and some review articles discuss its usage and limitations for solid-state ion diffusion [107,108]. Here, we will present a brief overview of quasielastic neutron scattering with examples.

**Figure 14:**
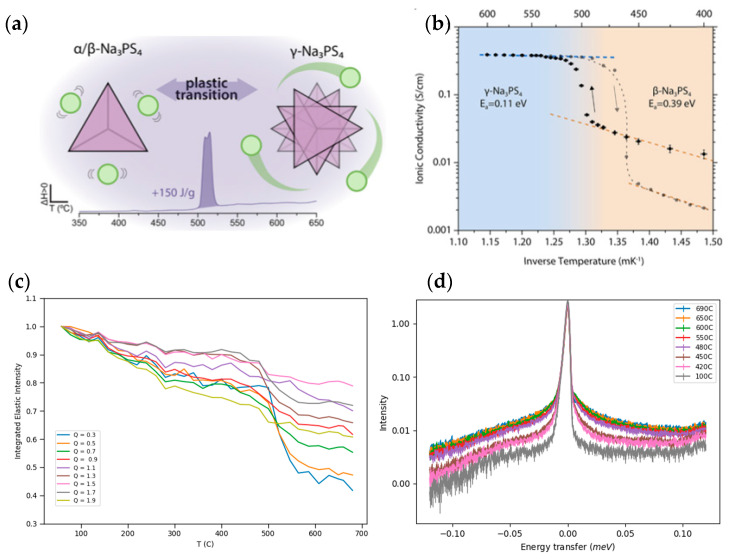
(**a**) Structural features of NaPS_4_ [133], (**b**) ionic conductivity of NaPS_4_ in both the β and γ phases [133], (**c**) elastic scan of Na_3_PS_4_, and (**d**) QENS spectra of Na_3_PS_4_ at temperatures from 100 to 690 °C illustrating different dynamical behavior for three different structural phases [135]. (**a**,**b**) Reprinted with permission from [133]. Copyright 2019 American Chemical Society.

A typical QENS spectrum of a solid-state electrolyte, Na_3_PS_4_, is shown in Figure 14d. The elastic peak is centered around zero energy transfer, with the width corresponding to the instrumental resolution. The instrumental resolution defines the accessible time scale for dynamic motions, which is a unique parameter for each quasielastic neutron spectrometer. Conceptually, two types of QENS spectrometers exist: time-of-flight spectrometers can access time scales from 10^−10^ to 10^−12^ s, while backscattering spectrometers can access somewhat slower motions with times from 10^−9^ to 10^−12^ s. If a dynamic process is observable on the time scale of the spectrometer, it will lead to a broadening of the elastic peak, which is the quasielastic signal. If the dynamic process is significantly slower than the instrumental energy resolution, the quasielastic signal will be too narrow and cannot be distinguished from the elastic peak. On the other hand, if the dynamics are much faster than the instrumental resolution, the quasielastic component will be a very broad, flat feature that cannot be distinguished from the background. Therefore, it is important to note that the characteristic times of the diffusive motions should be on the same order as the instrumental resolution. Elastic energy scans are a common method employed in QENS studies to locate the onset of observable dynamics and dynamic transitions. An elastic scan is performed by following the elastic intensity as a function of temperature by integrating the elastic peak. The elastic and quasielastic intensities are coupled, such that when a quasielastic process becomes observable at the measured time scale, the intensity of the elastic peak will decrease.

For solid materials, the quasielastic broadening can usually be modeled using Lorentzian functions, with a half-width at half-maxima (HWHM), Γ, that are inversely proportional to the residence time of the measured dynamic processes. While the quasielastic width is correlated to the characteristic time, the momentum transfer provides information about the nature and the length scales of the diffusive motion. By analyzing the Q-dependence of the quasielastic scattering and applying different theoretical models to fit the data, geometric and temporal information can be extracted, such as jump lengths, residence times, diffusion coefficients, activation energies, symmetry of motion, mean squared displacements, etc. Comparisons of these values to structural data help form a descriptive picture of the ionic diffusion mechanism on the atomic scale. For example, a jump length extracted from a jump diffusion model can be compared to the atomic distances in the crystal structure to determine the possible diffusion pathways and conduction mechanisms [109]. Diffusion coefficients and activation energies can be calculated to shed light on the energy landscape governing ionic transport. Also, reorientational and relaxational motions of the ionic framework can have significant effects on the conduction process, for example, by the paddle wheel mechanism [110]. In addition, the elastic incoherent structure factor (EISF), which represents the fraction of elastically scattered neutrons, provides insight into the volume that the particles are able to explore, hence revealing localized diffusion processes and reorientational hopping mechanisms [94].

The accessible length and time scales of QENS are comparable to the elementary dynamical processes of battery materials and solid-ionic conductors. Particularly in demand have been QENS experiments on solid-state lithium and sodium ion battery materials [111,112,113,114,115,116,117,118,119,120,121,122,123], but also alternative ionic conductors have been studied, such as Ag, Co, O, and Mg [124,125,126,127,128]. Lithium and sodium thiophosphates (Li_3_PS_4_ and Na_3_PS_4_) have been attracting attention as promising solid electrolytes for all-solid electrolyte batteries [129,130,131,132]. However, their ambient temperature ionic conductivities remain low, in the range of 10^−7^ to 10^−5^ S/cm, for practical applications. Structural phase transitions and substitution of P atoms have been seen as possible solutions in order to obtain higher ionic conductivities that would be more feasible for applications. Na_3_PS_4_ has three known polymorphs: the room-temperature tetragonal form (α), which transforms to a closely related cubic form (β) above 261 °C [130], and a high-temperature orthorhombic (γ) form above 517 °C [133]. The high-temperature phase of Na_3_PS_4_ exhibits fast Na^+^ conduction on the order of 10^−1^ S/cm and plastic crystal characteristics, including rotational motion of the PS_4_ anions [131,134]. These reorientational motions can have profound impacts on the ionic transport process in solid-state electrolytes. As Figure 14a illustrates, the anion’s rotational motions create more free volume for the cations to diffuse through, which reduces the energy barriers for diffusion and leads to faster, more efficient charge transport. The ionic conductivity for Na_3_PS_4_ for its β and γ phases is shown in Figure 14b [133], while the elastic scan was measured to estimate the Na ion dynamics, as shown in Figure 14c [135]. A clearly noticeable step in both the elastic intensity and the ionic conductivities is observed around the phase transition temperature (517 °C), indicating the onset of observable sodium ion dynamics at the measured timescale. The elastic intensity step is steeper for smaller Q values, which correspond to longer distances, while the higher Q values do not show such an obvious step. This indicates that the dynamic process is due to a long-range translational diffusion, with similar behavior seen for the ionic conductivity. The QENS spectra is shown in Figure 14d at temperatures ranging from 100 to 690 °C. Each phase (α, β, γ) shows a distinct QENS spectra, demonstrating very different dynamics taking place in each phase, with faster motions increasing the broadening of the elastic peak at higher temperatures. Furthermore, Gupta et al. investigated Na_3_PS_4_ using QENS and MLMD simulations, which showed that the Q-dependence of the motion obeyed the Chudley–Elliott jump diffusion model with a Na^+^ jump length of 3.6 Å [120]. The estimated diffusion coefficient, D, is on the order of ~10^−6^ cm^2^ s^−1^ at 600 K, which is typical for superionic conductors.

This section reviewed the powerful use of QENS for revealing the ionic conduction mechanism in solid-state battery materials. The fundamental motions of these materials on the atomic scale directly impact the overall functional performance of devices. Faster charging, high-efficiency batteries will require solid electrolytes with peak-performance characteristics and exhibiting fast ionic transport. QENS is one of the best available techniques for examining this conductivity process and will continue to help guide the optimization and development of novel batteries with improved solid-state electrolytes.

## 5. Future Outlook and Conclusions

Advanced batteries require advanced characterization techniques. This review gives an overview of the neutron scattering capabilities available at the moment. The sensitivity of neutrons to light elements, coupled with their penetrability through matter, remain attractive characteristics of neutron scattering in the study of battery materials. Some techniques that are unique to neutron scattering, e.g., quasielastic neutron scattering or the determination of the phonon density of states over the entire Brillouin zone with inelastic neutron scattering, will continue to support ionic transport mechanisms research for the foreseeable future. However, access to neutrons remains limited in comparison to other experimental techniques. Furthermore, neutron scattering is still an intensity limited technique compared to synchrotrons.

There is, however, reason for optimism. Four major neutron scattering facilities in operation (the Rutherford-Appleton Laboratory in the UK, the Institut Laue-Langevin in France, the J-PARC facility in Japan, and the Spallation Neutron Source/High Flux Isotope Reactor at ORNL in the US) offer access to an increasingly large number of beam lines implementing a growing array of techniques and sample environment while delivering high neutron flux. The European Spallation Source (ESS) [136,137], under construction in Lund, Sweden, and the planned Second Target Station [138] for the Spallation Neutron Source at SNS will further expand access while offering new capabilities, particularly regarding moderator brilliance, which will enable the use of much smaller samples. The ESS will start its user program in 2027 with the proton beam power slowly increasing to 2 MW over a period of a few years. It will become immediately competitive with the four sources mentioned above. Ultimately, ESS plans on operating at 5 MW, which will make it the most intense neutron source worldwide, likely by 2050. Additionally, new developments in neutron detection technology, neutron optics (e.g., neutron guides or focusing devices), static or dynamic collimators, wide-angle polarizers, faster electronics, and data acquisition systems, etc., will shorten data collection time, improve signal-to-background ratios, enable more complex experiments, and deliver more flux to users.

As far as new types of experiments are concerned, some trends are already discernible today. Access to high neutron flux makes it possible to perform kinetic (operando) measurements of physical or chemical processes on the time scale of minutes. Pulsed sources also permit one to consider pump-probe-type experiments with sample environment equipment synchronized with the accelerator heartbeat to observe transient physical or chemical phenomena. Overall, experiments are also becoming more complex. This trend has been apparent for over two decades and will continue unabated. The accessible range of traditional physical parameters, such as temperature, pressure, or magnetic field, will continue to expand, but new environmental conditions, including photoirradiation, electric field/high voltage, electrical/ionic conductivity, calorimetry, or impedance spectroscopy, are likely to become routinely available on multiple beam lines. First attempts are already occurring along these lines. Current planning of future neutron facilities now also includes multifunctional instruments implementing more than one scattering technique in any given beam line (for example, simultaneous diffraction and small-angle neutron scattering) but also the simultaneous use of multiple pieces of sample environment (for example, low-temperature inelastic neutron scattering at high pressure in a magnetic field). Increasing neutron flux and more complex experiments translate into a need for expanded data storage and data processing speed. The relevant technologies are already under development today. It is also conceivable that modeling of neutron data will run concurrently with the data acquisition at a beam line. Machine learning and artificial intelligence are developing at an accelerated pace and will play a role in data analysis and interpretation, particularly with respect to the more complex experiments.

It is not difficult to imagine how the scientific and technological advances described here will continue to benefit and advance battery research for the next few decades. The study of new electrolytes or anode and cathode materials, interfacial processes, ionic conduction mechanisms, battery degradation and lifespan, battery safety, performance, etc. Faster data acquisition, more complex experiments, better neutron beam time access, and accelerated data analysis will undoubtedly accelerate discoveries related to electrical energy storage. The future outlook for neutron-based research in the battery field is very positive.

In conclusion, we reviewed the current state of neutron scattering techniques for the investigation of battery materials, with a focus on solid-state materials and in situ/in operando measurements. Examples from neutron diffraction, total scattering, and pair distribution analysis highlighted the atomic-scale structural evolution of battery components during charge cycling. Small-angle neutron scattering and neutron reflectometry demonstrated the ability to probe larger (nanoscale to bulk) dimensions, including interfacial boundaries and the formation of the solid–electrolyte interface. Neutron imaging experiments were shown that examined the bulk-scale lithium transport and distribution, dendrite formation, cracking, delamination, and cell failure mechanisms, as well as advanced computed tomography techniques for virtual unrolling and slicing of electrode tomographs. Lastly, neutron spectroscopy, consisting of vibrational spectroscopy and quasielastic neutron scattering, showed the lattice dynamics, formation of the SEI, and the atomic nature of the ionic diffusion processes in solid electrolytes. Neutron scattering remains well positioned for the foreseeable future as a cutting-edge tool at the forefront of fundamental and applied research on advanced battery designs, specifically those employing solid-state components.

## Figures and Tables

**Table 1:** Refined thicknesses for the amorphous silicon and solid electrolyte interface layers [72].

Salt and Potential	aSi (Å)	SEI (Å)
In Air	365.0 ± 0.4	–
LiPF_6_ Open Circuit Voltage	376.2 ± 1.4	110.3 ± 2.9
LiPF_6_ 0.06 V	976.0 ± 11.8	167.6 ± 13.3
Exchange to LiBF_4_ 0.06 V	940.1 ± 10.8	115.0 ± 5.4
LiBF_4_ 1.2 V	422.5 ± 2.1	146.2 ± 3.6
LiBF_4_ 0.06 V 2nd Lithiation	1026.3 ± 7.0	206.9 ± 13.2

## Data Availability

No new data were created or analyzed in this study.
